# Effect of *TRIB1* Variant on Lipid Profile and Coronary Artery Disease: A Systematic Review and Meta-Analysis

**DOI:** 10.1155/2023/4444708

**Published:** 2023-01-09

**Authors:** Baozhu Wei, Yang Liu, Hang Li, Yuanyuan Peng, Zhi Luo

**Affiliations:** ^1^Department of Cardiology, Zhongnan Hospital of Wuhan University, Wuhan University, Wuhan, China; ^2^Institute of Myocardial Injury and Repair, Wuhan University, Wuhan, China; ^3^Department of Endocrinology, China Resources and WISCO General Hospital, Wuhan, China; ^4^Department of Geratology, Zhongnan Hospital of Wuhan University, Wuhan University, Wuhan, China

## Abstract

**Background:**

Emerging evidence indicates tribbles homolog 1 (Trib1) protein may be involved in lipid metabolism regulation and coronary artery disease (CAD) pathogenesis. However, whether *TRIB1* gene variants affect lipid levels and CAD remains elusive, this study is aimed at clarifying the effect of *TRIB1* variants on lipid profile and CAD.

**Methods:**

By searching PubMed and Cochrane databases for studies published before December 18, 2022, a total of 108,831 individuals were included for the analysis.

**Results:**

The outcomes of the analysis on all individuals showed that the A allele carriers of rs17321515 and rs2954029 variants had higher low-density lipoprotein cholesterol (LDL-C) and total cholesterol (TC) levels than the noncarriers. Consistently, a higher CAD risk was observed in the A allele carriers. Subgroup analysis indicated that increased LDL-C, TC, and CAD risk were observed in Asian population.

**Conclusions:**

Variants of *TRIB1* (i.e., rs17321515 and rs2954029) may serve as causal genetic markers for dyslipidemia and CAD in Asian population.

## 1. Introduction

Trib1, a serine-threonine kinase-like protein, encodes by the *TRIB1* gene, and it is proposed that Trib1 acts as an adaptor protein in multiple pathways, but the precise molecular function is unknown. Trib1 expression is ubiquitous and predominately in the liver [1] and coronary arteries [2]. Preliminary clinical data indicate that Trib1 expression is largely elevated in the coronary artery of advanced CAD [3].

It is now increasingly evidenced that Trib1 may be a promising regulator of lipid metabolism [4]. For instance, Trib1 knock-out increased mice's plasma triglycerides (TG) and cholesterol levels [5]. In contrast, overexpression of Trib1 reduced and normalized these parameters [6]. Moreover, the adenovirus-mediated rescue of Trib1 expression in liver-specific Bmal1 knock-out mice lowered plasma proprotein convertase subtilisin/kexin type 9 (PCSK9) levels, increased low-density lipoprotein receptor (LDLR) counts, and decreased plasma LDL-C levels [7]. However, deletion of Trib1 increased CCAAT/enhancer-binding protein alpha (CEBP*α*) and activated transcription factor 3 (ATF3) levels, reduced LDLR counts, and elevated plasma LDL-C levels [8]. Collectively, it indicated that Trib1 protein expression was closely related to lipid metabolism.

Currently, some functional variants of *TRIB1* altered mRNA secondary structure [9] therefore impacting Trib1 protein expression [6, 10]. Therefore, variants of rs2954029 and rs17321515 may also modulate the expression of Trib1.

Dyslipidemia is characterized by increased TG, TC, and LDL-C levels and decreased high-density lipoprotein cholesterol (HDL-C) levels in plasma. Since dyslipidemia is one of the most critical risk factors for CAD and accounts for at least 50% of population-attributable risk [11], it is tempting to speculate that the increased CAD risk caused by rs2954029 [12] and rs17321515 [13] variants may stem from atherogenic dyslipidemia. However, a series of GWAS identified rs2954029 [14–18], and rs17321515 [19] variant was associated only with higher TG levels in Caucasians. Two GWAS further indicated that the association of rs2954029 [20] and rs17321515 [21] variants with CAD was mediated by the elevated TG levels. However, whether variants of rs2954029 and rs17321515 were associated with other lipid parameters (e.g., LDL-C, TC, and HDL-C), it remains unknown. In order to clarify it, this study is conducted to clarify the association of rs17321515 and rs2954029 variants with lipid levels and CAD risk.

## 2. Methods

The present meta-analysis is in accordance with the Preferred Reporting Items for Systematic Reviews and Meta-Analyses (PRISMA) [22].

### 2.1. Literature Search

A comprehensive search of the literature was performed from June 1, 2021 to December 18, 2022, by using relevant databases including PubMed, Cochrane Library, Embase, Medline, Google Scholar, and Web of Science. The following keywords were used in the search: (“tribbles homolog 1,” “TRIB1,” “rs17321515,” or “rs2954029”), (“polymorphism,” “mutant,” “mutation,” “variant,” “variation,” “SNP,” or “single nucleotide polymorphism”) and (“lipid,” “plasma lipid,” “circulating lipid,” “serum lipid,” “blood lipid”).

### 2.2. Inclusion Criteria

The inclusion criteria for association analysis of variants of rs17321515 and rs2954029 with CAD were as follows: (1) studies using a population-based case-control design; (2) CAD cases were angiographically defined; (3) genotype frequencies of cases and controls according to variants of rs17321515 and rs2954029 were available. The inclusion criteria for association analysis of variants of rs17321515 and rs2954029 with lipid levels were as follows: (1) the studies investigated the association of TRIB1 rs17321515 or rs2954029 variants with lipid levels; (2) the studies at least provided one of four parameters in lipid profile (TG, TC, LDL-C, and HDL-C); (3) the studies provided the genotype frequencies of variants of rs17321515 and rs2954029; (4) the studies provided mean lipid levels with standard deviation (SD) or standard errors (SE) by the genotypes of rs17321515 and rs2954029; (5) the interventional studies provided preintervention data; (6) the language of eligible studies was restricted to English or Chinese.

### 2.3. Data Extraction

Data screening between the two authors was compared by kappa statistics [23]. Two authors extracted the data independently by using standardized data extraction tables. The discrepancy in data collected was resolved by consensus or a discussion with the third author. If key data was absent, e-mail or telephone was used to contact the corresponding authors to acquire this information. The following data were extracted from each eligible study: the first author's name, year, country, ethnicity, gender, genotype count, genotyping methods, type of study, type of disease, total sample size, and mean lipid levels with SD or SE by genotypes.

### 2.4. Data Analysis

The units of TG, TC, LDL-C, and HDL-C were converted into mmol/L. All extracted data were expressed as mean ± SD. The odds ratio (OR) with 95% confidence interval (CI) was used to evaluate the strength of variants of rs17321515 and rs2954029 with CAD risk. The standardized mean difference (SMD) and 95% CI were used to evaluate the differences in lipid levels between the genotypes rs17321515 and rs2954029. The pooled OR was performed for allelic model (A vs. G for rs17321515 and A vs. T for rs2954029), additive model (AA vs. GG for rs17321515 and AA vs. TT for rs2954029), dominant model ((GA+AA) vs. GG for rs17321515 and (TA+AA) vs. TT for rs2954029), and recessive model ((GG+GA) vs. AA for rs17321515 and (TT+TA) vs. AA for rs2954029). Since most of the included studies presented lipid data in a dominant model ((GA+AA) vs. GG for rs17321515 and (TA+AA) vs. TT for rs2954029), a dominant model was adopted to ensure adequate statistical power. All statistical tests were conducted with the Cochrane Collaboration meta-analysis software, Review Manager 5.4. *P* < 0.05 was recognized as statistically significant.

### 2.5. Subgroup Analysis

Subgroup analysis was carried out by ethnicity, gender, and the general population. Subgroup analysis by ethnicity was primarily conducted in the Asian cohort. Subgroup analysis by gender was performed in males and females. In some studies, the subjects were divided into more than one subpopulation (e.g., the subjects originated from different gender, case, and control subjects). Each subpopulation was regarded as an independent comparison in this study.

### 2.6. Other Analyses

Refer to the previous publication [24] for more details about heterogeneity processing, sensitivity analysis, risk bias test, and publication bias test.

## 3. Results

### 3.1. Study Selection

**Figure 1:**
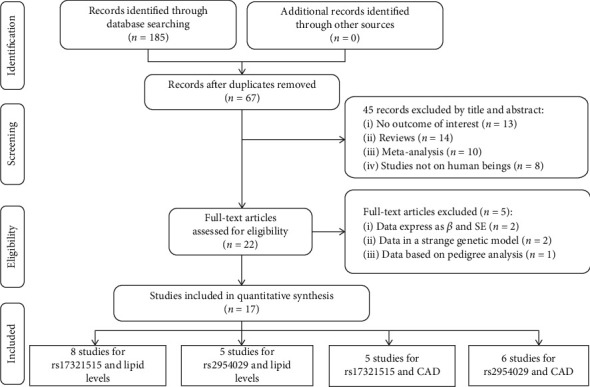
Flow diagram of the article's selection process.

The kappa value was 0.95 (>0.75) between the authors, and the details of the study selection were summarized in Figure 1 (please see Figure S1 for the full electronic search strategy).

### 3.2. Characteristics of Included Studies

The meta-analysis of *TRIB1* rs2954029 variant with lipid profile was presented in Supplementary Material: Table S1. The meta-analysis of *TRIB1* rs17321515 variant with CAD risk was presented in Supplementary Material: Table S2. The meta-analysis of *TRIB1* rs2954029 variant with CAD risk was presented in Supplementary Material: Table S3. The characteristics of the individual studies included in the meta-analysis between *TRIB1* variants and lipid profile were presented in Supplementary Material: Table S4. The characteristics of the individual studies included in the meta-analysis between *TRIB1* rs17321515 variant and CAD were presented in Supplementary Material: Table S5. The characteristics of the individual studies included in the meta-analysis between *TRIB1* rs2954029 variant and CAD were presented in Supplementary Material: Table S6. The plasma lipid levels by the genotypes of *TRIB1* rs17321515 variant were presented in Supplementary Material: Table S7. The plasma lipid levels by the genotypes of *TRIB1* rs2954029 variant were presented in Supplementary Material: Table S8.

### 3.3. Effect of rs17321515 on Lipid Profile

**Figure 2:**
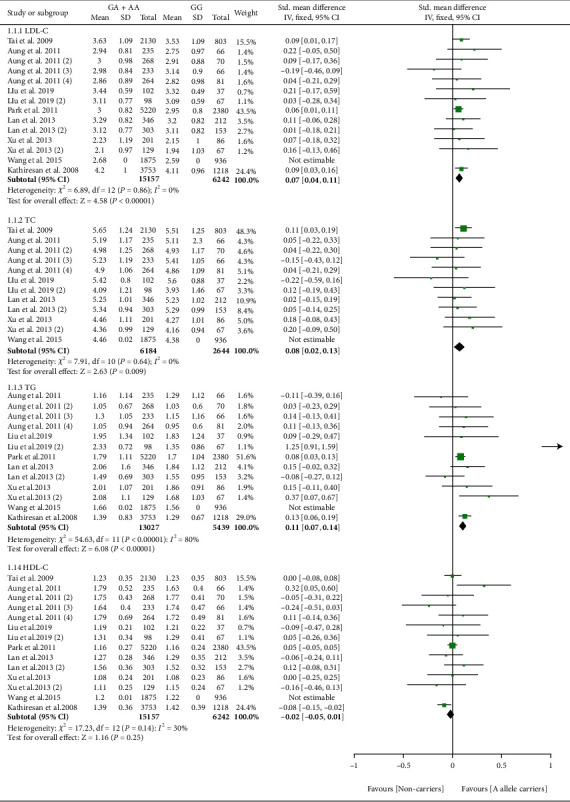
Forest plot of *TRIB1* rs17321515 variant with lipid profile.

The effect of rs17321515 on lipid profile was harmful (Figure 2). Subgroup analysis indicated that the significant effect of rs17321515 on lipid profile was primarily in Asians and the general population (please see Table 1 for more details).

### 3.4. Effect of rs2954029 on Lipid Profile

**Figure 3:**
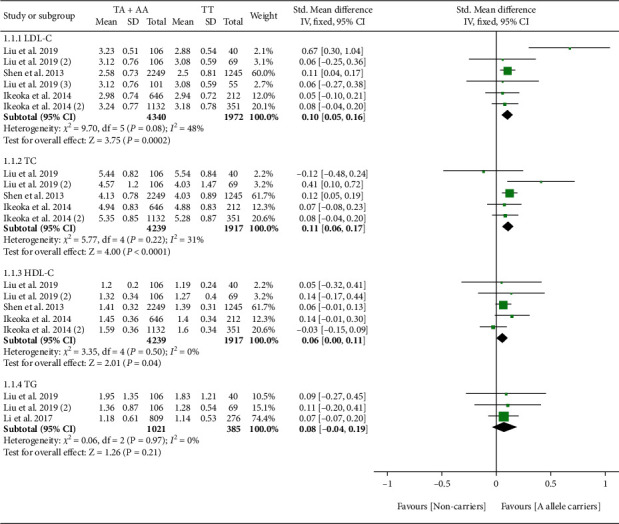
Forest plot of *TRIB1* rs2954029 variant with lipid profile.

rs2954029 had an ambiguous effect on lipid profile (Figure 3). Subgroup analysis indicated that the significant effect of rs2954029 on lipid profile was primarily in Asians and the general population (please see Table S1 for more details).

### 3.5. Effect of rs17321515 on CAD

**Figure 4:**
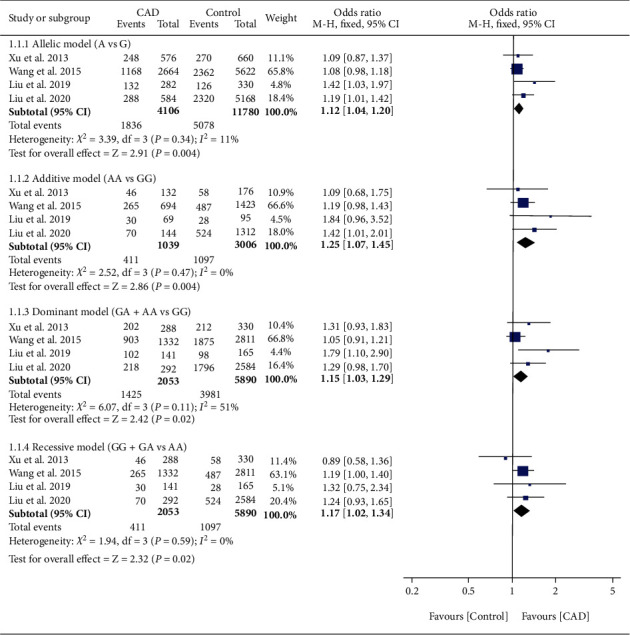
Forest plot of *TRIB1* rs17321515 variant with CAD risk.

The effect of rs17321515 on CAD was harmful (Figure 4). Subgroup analysis indicated that the effect of rs17321515 on CAD was observed in Asians (Table S2).

### 3.6. Effect of rs2954029 on CAD

**Figure 5:**
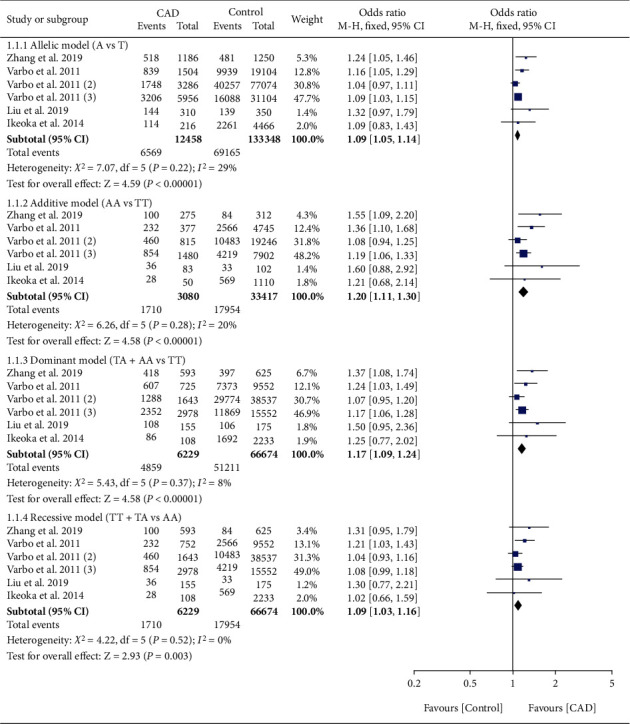
Forest plot of *TRIB1* rs2954029 variant with CAD risk.

The effect of rs2954029 on CAD was harmful (Figure 5). Subgroup analysis indicated that the effect of rs2954029 on CAD was observed in Asians and Caucasians (Table S3).

### 3.7. Evaluation of Heterogeneity

Significant heterogeneity was detected in analyzing the effect of rs17321515 and rs2954029 on CAD risk (Table S2, Table S3). Notably, the recalculated results changed substantially after eliminating heterogeneity (see Table S2 and Table S3 for more details).

### 3.8. Sensitivity Analysis

Sensitivity analysis indicated that some comparisons may influence the effect of rs17321515 and rs2954029 on lipid and CAD risk (please see Figure S2-S4 for more details). However, the effects of rs17321515 and rs2954029 on lipid and CAD did not change substantially after omitting these comparisons, indicating that the synthetic results were robust.

### 3.9. Risk Bias Test

The effects of rs17321515 and rs2954029 on lipid and CAD showed a low risk of bias (see Figure S5 for more details), indicating that the included studies were of relatively high quality.

### 3.10. Publication Bias Test

Begg's test did not find any publication bias in the present study, which was confirmed by Egger's regression test (see Figure S6-S9 for more details).

## 4. Discussion

The present study showed that rs17321515 and rs2954029 caused atherogenic dyslipidemia and increased CAD risk in Asians, indicating that the Asian populations were at high risk of CAD.

Previous studies indicated that inhibition of Trib1 caused atherogenic dyslipidemia [8], while overexpression [6] or rescue [7] of Trib1 remodeled lipid metabolism homeostasis. Therefore, rs2954029 and rs17321515 may affect lipid levels by influencing Trib1 expression [6, 9, 10].

The present study showed that rs17321515 increased LDL-C, TC, and TG levels (Table 1). Since dyslipidemia played a critical role in CAD pathogenesis [11], it indicated that increased CAD risk associated with rs17321515 (Table S2) was mediated, at least partly, by the increased LDL-C, TC, and TG levels (Table 1). In contrast, rs2954029 increased LDL-C (harmful), TC (harmful), and HDL-C (beneficial) levels (Table S1), indicating that rs2954029 had an ambiguous effect on lipid profile. When combined with Shihab et al.'s [11] study, it indicated that the increased CAD risk associated with rs2954029 (Table S3) was mediated by the increased LDL-C and TC levels (Table S1).

According to the 2018 ACC/AHA [25], the 2019 ESC/EAS [26], and the adult treatment panel III (ATP III) cholesterol guidelines [27], LDL-C was considered the major cause of CAD and treated as the primary target for therapy, while other lipids were used as the secondary or supplementary therapeutic targets. In the present study, significantly increased LDL-C levels were observed in subjects with rs2954029 and rs17321515 (Table 1, Table S1), indicating that rs2954029 and rs17321515 may serve as causal genetic markers for dyslipidemia or CAD.

Subgroup analysis by ethnicity indicated that significantly increased LDL-C and TC were observed in Asians with rs2954029 and rs17321515 (Table 1, Table S1), indicating that Asians with rs2954029 and rs17321515 were at high risk of CAD. Intriguingly, this speculation was verified in the present study, whereas rs2954029 and rs17321515 significantly increased the risk of CAD in Asians (Table S2, Table S3). Meanwhile, rs2954029 significantly increased the risk of CAD in Caucasians (Table S3), indicating that Caucasians with rs2954029 had a high risk to develop CAD. However, whether rs17321515 impacted the risk of CAD in Caucasians could not be determined due to the absence of original data. Therefore, further clinical trials on Caucasians are certainly needed.

Subgroup analysis by gender indicated that rs17321515 did not show statistically significant effect on lipid profile in both males and females. However, only 3 comparisons (1158 individuals for males and 1139 individuals for females) were used to calculate the results in males and females (Table 1), which lowers the strength of the results and needs to be confirmed by future studies. Moreover, the effects of rs2954029 and rs17321515 on lipid and CAD were significant in general population, indicating that general population with rs2954029 and rs17321515 were at high risk of dyslipidemia or CAD.

### 4.1. Strengths and Limitations

The present meta-analysis has several strengths. For instance, the clinical data of 108,831 individuals were included, which increased the reliability of synthetic results due to high statistical power [28]. Moreover, the synthetic results were recalculated after excluding the studies with heterogeneity, which further advanced the preciseness of conclusions drawn in this manuscript and were not likely to be type I errors (false-positive results) [28]. However, several limitations of the present study should be noted. Firstly, a large number of genes and some environmental factors are involved in dyslipidemia and CAD [28]. Our study has not investigated the interaction of *TRIB1* variants with other variant loci or environmental factors on lipid profile and CAD risk due to the lack of original data from the included studies. In other words, more precise results could have been gained if more detailed individual data were available, or if the stratification analyses based on the environmental factors such as smoking, alcohol consumption, and exercise were performed [29]. Secondly, this meta-analysis only included the studies published in English and Chinese as it was very difficult to get the full papers published in various languages [29]. Thirdly, we did not register a protocol (e.g., PROSPERO) for this meta-analysis, which may introduce potential bias to this review.

## 5. Conclusions

Variants of *TRIB1* (i.e., rs17321515 and rs2954029) may serve as causal genetic markers for dyslipidemia and CAD in Asian population.

## Figures and Tables

**Table 1:** Meta-analysis of *TRIB1* rs17321515 variant with lipid profile.

Groups or subgroups	Comparisons (subjects)	P_H_	SMD (95% CI)	P_SMD_
TG				
All	13 (18 466)	<0.001	0.11 (0.07-0.14)	<0.001
Studies in HWE	11 (17 672)	<0.001	0.20 (0.08-0.32)	<0.01
Asian	12 (13 495)	<0.001	0.18 (0.03-0.32)	0.02
Male	3 (1 158)	0.25	0.08 (-0.08-0.24)	0.32
Female	3 (1 139)	0.48	0.00 (-0.13-0.13)	0.94
General population	10 (15 229)	<0.001	0.17 (0.05-0.29)	0.01
TC				
All	12 (8 828)	0.64	0.08 (0.02-0.13)	0.01
Studies in HWE	10 (8 034)	0.45	0.08 (0.02-0.14)	0.01
Asian	12 (8 828)	0.63	0.08 (0.02-0.13)	0.01
Male	3 (1 158)	0.50	-0.01 (-0.14-0.12)	0.87
Female	3 (1 139)	0.99	0.04 (-0.09-0.18)	0.50
General population	9 (5 591)	0.76	0.08 (0.02-0.14)	0.01
LDL-C				
All	14 (21 399)	0.86	0.07 (0.04-0.11)	<0.001
Studies in HWE	12 (20 605)	0.77	0.08 (0.04-0.11)	<0.001
Asian	13 (16 428)	0.83	0.07 (0.03-0.11)	<0.001
Male	3 (1 158)	0.09	0.07(-0.06-0.20)	0.29
Female	3 (1 139)	0.89	0.04 (-0.09-0.17)	0.53
General population	11 (18 162)	0.78	0.07 (0.04-0.11)	<0.001
HDL-C				
All	14 (21 399)	0.14	-0.02 (-0.05-0.01)	0.25
Studies in HWE	12 (20 605)	0.12	-0.02 (-0.05-0.01)	0.18
Asian	13 (16 428)	0.33	0.00 (-0.04-0.04)	0.94
Male	3 (1 158)	0.01	-0.02 (-0.15-0.11)	0.78
Female	3 (1 139)	0.59	0.07 (-0.06-0.21)	0.29
General population	11 (18 162)	0.07	-0.02 (-0.05-0.01)	0.26

SMD: standardized mean difference; 95% CI: 95% confidence interval; P_H:_ P_Heterogeneity;_ HWE: Hardy-Weinberg equilibrium; TG: triglycerides; TC: total cholesterol; LDL-C: low-density lipoprotein cholesterol; HDL-C: high-density lipoprotein cholesterol.

## Data Availability

All data used to support the findings of this study are included within the article and its supplementary materials.

## Supplementary Materials

Supplementary materials Table S1: meta-analysis of *TRIB1* rs2954029 variant with lipid profile. Table S2: meta-analysis of *TRIB1* rs17321515 variant with CAD risk. Table S3: meta-analysis of *TRIB1* rs2954029 variant with CAD risk. Table S4: characteristics of the individual studies included in the meta-analysis between *TRIB1* variants and lipid profile. Table S5: characteristics of the individual studies included in the meta-analysis between *TRIB1* rs17321515 variant and CAD. Table S6: characteristics of the individual studies included in the meta-analysis between *TRIB1* rs2954029 variant and CAD. Table S7: plasma lipid levels by the genotypes of *TRIB1* rs17321515 variant. Table S8: plasma lipid levels by the genotypes of *TRIB1* rs2954029 variant. Figure S1: the full electronic search strategy in PubMed. Figure S2: sensitivity analysis of *TRIB1* rs17321515 variant with lipid profile. Figure S3: sensitivity analysis of *TRIB1* rs17321515 variant with CAD risk. Figure S4: sensitivity analysis of *TRIB1* rs2954029 variant with CAD risk. Figure S5: risk bias plot of *TRIB1* variant with LDL-C levels and CAD risk. Figure S6. Begg's funnel plot of *TRIB1* rs17321515 variant with lipid profile. Figure S7: Begg's funnel plot of *TRIB1* rs2954029 variant with lipid profile. Figure S8: Begg's funnel plot of *TRIB1* rs17321515 variant with CAD risk. Figure S9: Begg's funnel plot of *TRIB1* rs2954029 variant with CAD risk.
