# Supplementary material for: Effect of TRIB1 Variant on Lipid Profile and Coronary Artery Disease: A Systematic Review and Meta-Analysis
Source: Cardiovasc Ther. 2023 Jan 9;2023:4444708. doi: 10.1155/2023/4444708 (PMC9842430; doi:10.1155/2023/4444708)
Supplement: Supplementary materials — Table S1: meta-analysis of TRIB1 rs2954029 variant with lipid profile. Table S2: meta-analysis of TRIB1 rs17321515 variant with CAD risk. Table S3: meta-analysis of TRIB1 rs2954029 variant with CAD risk. Table S4: characteristics of the individual studies included in the meta-analysis between TRIB1 variants and lipid profile. Table S5: characteristics of the individual studies included in the meta-analysis between TRIB1 rs17321515 variant and CAD. Table S6: characteristics of the individual studies included in the meta-analysis between TRIB1 rs2954029 variant and CAD. Table S7: plasma lipid levels by the genotypes of TRIB1 rs17321515 variant. Table S8: plasma lipid levels by the genotypes of TRIB1 rs2954029 variant. Figure S1: the full electronic search strategy in PubMed. Figure S2: sensitivity analysis of TRIB1 rs17321515 variant with lipid profile. Figure S3: sensitivity analysis of TRIB1 rs17321515 variant with CAD risk. Figure S4: sensitivity analysis of TRIB1 rs2954029 variant with CAD risk. Figure S5: risk bias plot of TRIB1 variant with LDL-C levels and CAD risk. Figure S6. Begg's funnel plot of TRIB1 rs17321515 variant with lipid profile. Figure S7: Begg's funnel plot of TRIB1 rs2954029 variant with lipid profile. Figure S8: Begg's funnel plot of TRIB1 rs17321515 variant with CAD risk. Figure S9: Begg's funnel plot of TRIB1 rs2954029 variant with CAD risk. [file 4444708.f1.doc]

**Supplemental Tables:**

**Table S1.** Meta-analysis of *TRIB1* rs2954029 variant with lipid profile.

**Table S2.** Meta-analysis of *TRIB1* rs17321515 variant with CAD risk.

**Table S3.** Meta-analysis of *TRIB1* rs2954029 variant with CAD risk.

**Table S4.** Characteristics of the individual studies included in the meta-analysis between *TRIB1* variants and lipid profile.

**Table S5.** Characteristics of the individual studies included in the meta-analysis between *TRIB1* rs17321515 variant and CAD.

**Table S6.** Characteristics of the individual studies included in the meta-analysis between *TRIB1* rs2954029 variant and CAD.

**Table S7.** Plasma lipid levels by the genotypes of *TRIB1* rs17321515 variant.

**Table S8.** Plasma lipid levels by the genotypes of *TRIB1* rs2954029 variant.

**Supplemental Figures:**

**Figure S1.** The full electronic search strategy in Pubmed.

**Figure S2.** Sensitivity analysis of *TRIB1* rs17321515 variant with lipid profile.

**Figure S3.** Sensitivity analysis of *TRIB1* rs17321515 variant with CAD risk.

**Figure S4.** Sensitivity analysis of *TRIB1* rs2954029 variant with CAD risk.

**Figure S5.** Risk bias plot of *TRIB1* variant with LDL-C levels and CAD risk.

**Figure S6.** Begg’s funnel plot of *TRIB1* rs17321515 variant with lipid profile.

**Figure S7.** Begg’s funnel plot of *TRIB1* rs2954029 variant with lipid profile.

**Figure S8.** Begg’s funnel plot of *TRIB1* rs17321515 variant with CAD risk.

**Figure S9.** Begg’s funnel plot of *TRIB1* rs2954029 variant with CAD risk.

**Table S1. Meta-analysis of *TRIB1* rs2954029 variant with lipid profile.**

| **Groups or subgroups** | **Comparisons (Subjects)** | ***P*_H_** | **SMD (95% CI)** | ***P*_SMD_** |
| --- | --- | --- | --- | --- |
| **TG** |  |  |  |  |
| All | 3 (1 406) | 0.97 | 0.08 (-0.04-0.19)  04 | 0.21 |
| Studies in HWE | 3 (1 406) | 0.97 | 0.08 (-0.04-0.19)  04 | 0.21 |
| Asian | 3 (1 406) | 0.97 | 0.08 (-0.04-0.19)  04 | 0.21 |
| General population | 2 (1 260) | 0.82 | 0.07 (-0.05-0.20) | 0.24 |
| **TC** |  |  |  |  |
| All | 5 (6 156) | 0.22 | 0.11 (0.06-0.17)  04 | <0.001 |
| Studies in HWE | 5 (6 156) | 0.22 | 0.11 (0.06-0.17)  04 | <0.001 |
| Asian | 5 (6 156) | 0.22 | 0.11 (0.06-0.17)  04 | <0.001 |
| General population | 4 (6 010) | 0.24 | 0.12 (0.06-0.17) | <0.001 |
| **LDL-C** |  |  |  |  |
| All | 6 (6 312) | 0.08 | 0.10 (0.05-0.16)  04 | <0.001 |
| Studies in HWE | 6 (6 312) | 0.08 | 0.10 (0.05-0.16)  04 | <0.001 |
| Asian | 6 (6 312) | 0.08 | 0.10 (0.05-0.16)  04 | <0.001 |
| General population | 4 (6 010) | 0.93 | 0.09 (0.04-0.15) | <0.01 |
| **HDL-C** |  |  |  |  |
| All | 5 (6 156) | 0.50 | 0.06 (0.00-0.11)  04 | 0.04 |
| Studies in HWE | 5 (6 156) | 0.50 | 0.06 (0.00-0.11)  04 | 0.04 |
| Asian | 5 (6 156) | 0.50 | 0.06 (0.00-0.11)  04 | 0.04 |
| General population | 4 (6 010) | 0.34 | 0.06 (0.00-0.11) | 0.05 |

SMD: standardized mean difference; 95% CI: 95% confidence interval; *P*_H_: *P*_Heterogeneity_; HWE: Hardy-Weinberg equilibrium; TG: triglycerides; TC: total cholesterol; LDL-C: low-density lipoprotein cholesterol; HDL-C: high-density lipoprotein cholesterol.

**Table S2. Meta-analysis of *TRIB1* rs17321515 variant with CAD risk.**

| **Groups or subgroups** | **Comparisons**  **(Subjects)** | ***P*_H_** | **OR (95% CI)** | ***P*_OR_** |  | **Groups or subgroups** | **Comparisons**  **(Subjects)** | ***P*_H_** | **OR (95% CI)** | ***P*_OR_** |
| --- | --- | --- | --- | --- | --- | --- | --- | --- | --- | --- |
| ***Overall results*** | | | | |  | ***Recalculated results that eliminated heterogeneity*** | | | | |
| **Allelic model (A vs G)** |  |  |  |  |  | **Allelic model (A vs G)** |  |  |  |  |
| All | 6 (14 691) | <0.01 | 1.05 (0.92-1.19)  04 | 0.50 |  | All | 4 (7 943) | 0.34 | 1.12 (1.04-1.20)  04 | <0.01 |
| Studies in HWE | 6 (14 691) | <0.01 | 1.05 (0.92-1.19)  04 | 0.50 |  | Studies in HWE | 4 (7 943) | 0.34 | 1.12 (1.04-1.20)  04 | <0.01 |
| Asian | 6 (14 691) | <0.01 | 1.05 (0.92-1.19)  04 | 0.50 |  | Asian | 4 (7 943) | 0.34 | 1.12 (1.04-1.20)  04 | <0.01 |
| **Additive model (AA vs GG)** |  |  |  |  |  | **Additive model (AA vs GG)** |  |  |  |  |
| All | 6 (14 691) | 0.01 | 1.08 (0.85-1.37)  04 | 0.55 |  | All | 4 (7 943) | 0.47 | 1.25 (1.07-1.45)  04 | <0.01 |
| Studies in HWE | 6 (14 691) | 0.01 | 1.08 (0.85-1.37)  04 | 0.55 |  | Studies in HWE | 4 (7 943) | 0.47 | 1.25 (1.07-1.45)  04 | <0.01 |
| Asian | 6 (14 691) | 0.01 | 1.08 (0.85-1.37)  04 | 0.55 |  | Asian | 4 (7 943) | 0.47 | 1.25 (1.07-1.45)  04 | <0.01 |
| **Dominant model (GA+AA vs GG )** |  |  |  |  |  | **Dominant model (GA+AA vs GG )** |  |  |  |  |
| All | 6 (14 691) | <0.01 | 1.08 (0.89-1.31)  04 | 0.43 |  | All | 4 (7 943) | 0.11 | 1.15 (1.03-1.29)  04 | 0.02 |
| Studies in HWE | 6 (14 691) | <0.01 | 1.08 (0.89-1.31)  04 | 0.43 |  | Studies in HWE | 4 (7 943) | 0.11 | 1.15 (1.03-1.29)  04 | 0.02 |
| Asian | 6 (14 691) | <0.01 | 1.08 (0.89-1.31)  04 | 0.43 |  | Asian | 4 (7 943) | 0.11 | 1.15 (1.03-1.29)  04 | 0.02 |
| **Recessive model (GG+GA vs AA)** |  |  |  |  |  | **Recessive model (GG+GA vs AA)** |  |  |  |  |
| All | 6 (14 691) | 0.59 | 1.17 (1.02-1.34)  04 | 0.02 |  | All | 6 (14 691) | 0.59 | 1.17 (1.02-1.34)  04 | 0.02 |
| Studies in HWE | 6 (14 691) | 0.59 | 1.17 (1.02-1.34)  04 | 0.02 |  | Studies in HWE | 6 (14 691) | 0.59 | 1.17 (1.02-1.34)  04 | 0.02 |
| Asian | 6 (14 691) | 0.59 | 1.17 (1.02-1.34)  04 | 0.02 |  | Asian | 6 (14 691) | 0.59 | 1.17 (1.02-1.34)  04 | 0.02 |

OR: odds ratio; 95% CI: 95% confidence interval; *P*_H_: *P*_Heterogeneity_; HWE: Hardy-Weinberg equilibrium; TG: triglycerides; TC: total cholesterol; LDL-C: low-density lipoprotein cholesterol; HDL-C: high-density lipoprotein cholesterol.

**Table S3. Meta-analysis of *TRIB1* rs2954029 variant with CAD risk.**

| **Groups or subgroups** | **Comparisons**  **(Subjects)** | ***P*_H_** | **OR (95% CI)** | ***P*_OR_** |  | **Groups or subgroups** | **Comparisons**  **(Subjects)** | ***P*_H_** | **OR (95% CI)** | ***P*_OR_** |
| --- | --- | --- | --- | --- | --- | --- | --- | --- | --- | --- |
| ***Overall results*** | | | | |  | ***Recalculated results that eliminated heterogeneity*** | | | | |
| Allelic model (A vs T) |  |  |  |  |  | **Allelic model (A vs T)** |  |  |  |  |
| All | 8 (73 832) | <0.01 | 1.06 (0.96-1.16)  04 | 0.24 |  | All | 6 (72 903) | 0.22 | 1.09 (1.05-1.14)  04 | <0.001 |
| Studies in HWE | 8 (73 832) | <0.01 | 1.06 (0.96-1.16)  04 | 0.24 |  | Studies in HWE | 6 (72 903) | 0.22 | 1.09 (1.05-1.14)  04 | <0.001 |
| Asian | 4 (4 544) | <0.01 | 1.07 (0.83-1.38) | 0.62 |  | Asian | 3 (3 889) | 0.63 | 1.22 (1.07-1.38)  04 | <0.01 |
| Caucasian | 3 (69 014) | 0.21 | 1.09 (1.03-1.14) | <0.01 |  | Caucasian | 3 (69 014) | 0.21 | 1.08 (1.04-1.13) | <0.001 |
| Additive model (AA vs TT) |  |  |  |  |  | **Additive model (AA vs TT)** |  |  |  |  |
| All | 8 (73 832) | <0.01 | 1.12 (0.94-1.35)  04 | 0.21 |  | All | 6 (72 903) | 0.28 | 1.20 (1.11-1.30)  04 | <0.001 |
| Studies in HWE | 8 (73 832) | <0.01 | 1.12 (0.94-1.35)  04 | 0.21 |  | Studies in HWE | 6 (72 903) | 0.28 | 1.20 (1.11-1.30)  04 | <0.001 |
| Asian | 4 (4 544) | 0.01 | 1.14 (0.69-1.87)  04 | 0.61 |  | Asian | 3 (3 889) | 0.74 | 1.48 (1.13-1.93)  04 | <0.01 |
| Caucasian | 3 (69 014) | 0.22 | 1.18 (1.06-1.31) | <0.01 |  | Caucasian | 3 (69 014) | 0.22 | 1.18 (1.09-1.28) | <0.001 |
| Dominant model (TA+AA vs TT) |  |  |  |  |  | **Dominant model (TA+AA vs TT)** |  |  |  |  |
| All | 8 (73 832) | <0.01 | 1.11 (0.96-1.28)  04 | 0.15 |  | All | 6 (72 903) | 0.37 | 1.17 (1.09-1.24)  04 | <0.001 |
| Studies in HWE | 8 (73 832) | <0.01 | 1.11 (0.96-1.28)  04 | 0.15 |  | Studies in HWE | 6 (72 903) | 0.37 | 1.17 (1.09-1.24)  04 | <0.001 |
| Asian | 4 (4 544) | 0.01 | 1.13 (0.77-1.66)  04 | 0.53 |  | Asian | 3 (3 889) | 0.87 | 1.37 (1.13-1.67)  04 | <0.01 |
| Caucasian | 3 (69 014) | 0.35 | 1.14 (1.06-1.23) | <0.001 |  | Caucasian | 3 (69 014) | 0.35 | 1.14 (1.07-1.22) | <0.001 |
| Recessive model (TT+TA vs AA) |  |  |  |  |  | **Recessive model (TT+TA vs AA)** |  |  |  |  |
| All | 8 (73 832) | 0.07 | 1.07 (0.96-1.19)  04 | 0.23 |  | All | 7 (73 558) | 0.52 | 1.09 (1.03-1.16)  04 | <0.01 |
| Studies in HWE | 8 (73 832) | 0.07 | 1.07 (0.96-1.19)  04 | 0.23 |  | Studies in HWE | 7 (73 558) | 0.52 | 1.09 (1.03-1.16)  04 | <0.01 |
| Asian | 4 (4 544) | 0.13 | 1.08 (0.88-1.33)  04 | 0.44 |  | Asian | 4 (4 544) | 0.13 | 1.08 (0.88-1.33)  04 | 0.44 |
| Caucasian | 3 (69 014) | 0.29 | 1.09 (1.02-1.16) | 0.01 |  | Caucasian | 3 (69 014) | 0.29 | 1.09 (1.02-1.16) | 0.01 |

OR: odds ratio; 95% CI: 95% confidence interval; *P*_H_: *P*_Heterogeneity_; HWE: Hardy-Weinberg equilibrium; TG: triglycerides; TC: total cholesterol; LDL-C: low-density lipoprotein cholesterol; HDL-C: high-density lipoprotein cholesterol.

**Table S4.** Characteristics of the individual studies included in the meta-analysis between variants of *TRIB1* and lipid profile.

| **First author, reference** | **Year** | **Country** | **Ethnicity** | **Gender** | **Study population** | **Outcomes** |
| --- | --- | --- | --- | --- | --- | --- |
| Tai et al. 2009 [R1] | 2009 | Singapore | Asian | M/F | General population | TC/LDL-C/HDL-C |
| Aung et al. 2011 [R2] | 2011 | China | Asian | M | General population | TG/TC/LDL-C/HDL-C |
| Aung et al. 2011 (2) [R2] | 2011 | China | Asian | F | General population | TG/TC/LDL-C/HDL-C |
| Aung et al. 2011 (3) [R2] | 2011 | China | Asian | M | General population | TG/TC/LDL-C/HDL-C |
| Aung et al. 2011 (4) [R2] | 2011 | China | Asian | F | General population | TG/TC/LDL-C/HDL-C |
| Liu et al. 2019 [R3] | 2019 | China | Asian | M/F | Patients with NAFLD | TG/TC/LDL-C/HDL-C |
| Liu et al. 2019 (2) [R3] | 2019 | China | Asian | M/F | General population | TG/TC/LDL-C/HDL-C |
| Park et al. 2011 [R4] | 2011 | South Korea | Asian | M/F | General population | TG/LDL-C/HDL-C |
| Lan et al. 2013 [R5] | 2013 | China | Asian | M | General population | TG/TC/LDL-C/HDL-C |
| Lan et al. 2013 (2) [R5] | 2013 | China | Asian | F | General population | TG/TC/LDL-C/HDL-C |
| Xu et al. 2013 [R6] | 2013 | China | Asian | M/F | Patients with CAD | TG/TC/LDL-C/HDL-C |
| Xu et al. 2013 (2) [R6] | 2013 | China | Asian | M/F | General population | TG/TC/LDL-C/HDL-C |
| Wang et al. 2015 [R7] | 2015 | China | Asian | M/F | Patients with CAD | TG/TC/LDL-C/HDL-C |
| Kathiresan et al. 2008 [R8] | 2008 | USA | Caucasion | M/F | General population | TG/LDL-C/HDL-C |
| Shen et al. 2013 [R11] | 2013 | China | Asian | M/F | General population | TC/LDL-C/HDL-C |
| Liu et al. 2019 (3) [R12] | 2019 | China | Asian | M/F | Patients with CAD | LDL-C |
| Ikeoka et al. 2014 [R13] | 2014 | Japan | Asian | M | Patients with CAD | TC/LDL-C/HDL-C |
| Ikeoka et al. 2014 (2) [R13] | 2014 | Japan | Asian | F | Patients with CAD | TC/LDL-C/HDL-C |
| Li et al. 2017 [R14] | 2017 | China | Asian | M/F | General population | TG |

*TRIB1:* tribbles homolog 1; M: male; F: female; CAD: coronary artery disease; NAFLD: Non-alcoholic fatty liver disease; TG: triglycerides; TC: total cholesterol; LDL-C: low-density lipoprotein cholesterol; HDL-C: high-density lipoprotein cholesterol.

**Table S5.** Characteristics of the individual studies included in the meta-analysis between *TRIB1* rs17321515 variant and CAD.

| **First author, reference** | **Year** | **Country** | **Ethnicity** | **Gender** | **Sample size**  **(case/control)** | **Case (N)** | | |  | **Control (N)** | | | ***P*_HWE_** |
| --- | --- | --- | --- | --- | --- | --- | --- | --- | --- | --- | --- | --- | --- |
|  |  |  |  |  |  | **GG** | **GA** | **AA** |  | **GG** | **GA** | **AA** |  |
| Xu et al. 2013 [R6] | 2013 | China | Asian | M/F | 288/330 | 86 | 156 | 46 |  | 118 | 154 | 58 | 0.53 |
| Wang et al. 2015 [R7] | 2015 | China | Asian | M/F | 1332/2811 | 429 | 638 | 265 |  | 936 | 1388 | 487 | 0.48 |
| Liu et al. 2020 [R9] | 2020 | China | Asian | M/F | 824/5269 | 261 | 396 | 167 |  | 1574 | 2564 | 1131 | 0.15 |
| Liu et al. 2020 (2) [R9] | 2020 | China | Asian | M/F | 292/2584 | 74 | 148 | 70 |  | 788 | 1272 | 524 | 0.80 |
| Cai et al. 2017 [R10] | 2017 | China | Asian | M/F | 162/493 | 60 | 76 | 26 |  | 137 | 249 | 107 | 0.76 |
| Liu et al. 2019 (3) [R12] | 2019 | China | Asian | M/F | 141/165 | 39 | 72 | 30 |  | 67 | 70 | 28 | 0.19 |

*TRIB1:* tribbles homolog 1; CAD: coronary artery disease; HWE: Hardy-Weinberg equilibrium.

**Table S6.** Characteristics of the individual studies included in the meta-analysis between *TRIB1* rs2954029 variant and CAD.

| **First author, reference** | **Year** | **Country** | **Ethnicity** | **Gender** | **Sample size**  **(case/control)** | **Case (N)** | | |  | **Control (N)** | | | ***P*_HWE_** |
| --- | --- | --- | --- | --- | --- | --- | --- | --- | --- | --- | --- | --- | --- |
|  |  |  |  |  |  | **TT** | **TA** | **AA** |  | **TT** | **TA** | **AA** |  |
| Cai et al. 2017 [R10] | 2017 | China | Asian | M/F | 162/493 | 61 | 75 | 26 |  | 138 | 247 | 108 | 0.90 |
| Liu et al. 2019 (3) [R12] | 2019 | China | Asian | M/F | 155/175 | 47 | 72 | 36 |  | 69 | 73 | 33 | 0.09 |
| Ikeoka et al. 2014 [R13] | 2014 | Japan | Asian | M/F | 108/2233 | 22 | 58 | 28 |  | 541 | 1123 | 569 | 0.78 |
| Zhang et al. 2019 [R15] | 2019 | China | Asian | M/F | 593/625 | 175 | 318 | 100 |  | 228 | 313 | 84 | 0.15 |
| Varbo et al. 2011 [R16] | 2011 | Denmark | Caucasian | M/F | 752/9552 | 145 | 375 | 232 |  | 2179 | 4807 | 2566 | 0.43 |
| Varbo et al. 2011 (2) [R16] | 2011 | Denmark | Caucasian | M/F | 1643/38537 | 355 | 828 | 460 |  | 8763 | 19291 | 10483 | 0.53 |
| Varbo et al. 2011 (3) [R16] | 2011 | Denmark | Caucasian | M/F | 2978/15552 | 626 | 1498 | 854 |  | 3683 | 7650 | 4219 | 0.06 |
| Karimi et al. 2022 [R17] | 2022 | Iran | Iranian | M/F | 125/149 | 23 | 66 | 36 |  | 14 | 75 | 60 | 0.17 |

*TRIB1:* tribbles homolog 1; CAD: coronary artery disease; HWE: Hardy-Weinberg equilibrium.

**Table S7.** Plasma lipid levels by the genotypes of *TRIB1* rs17321515 variant.

| **First author, reference** | **Number** | |  | **TG, mmol/L** | |  | **TC, mmol/L** | |  | **LDL-C, mmol/L** | |  | **HDL-C, mmol/L** | |
| --- | --- | --- | --- | --- | --- | --- | --- | --- | --- | --- | --- | --- | --- | --- |
|  | **GG** | **GA+AA** |  | **GG** | **GA+AA** |  | **GG** | **GA+AA** |  | **GG** | **GA+AA** |  | **GG** | **GA+AA** |
| Tai et al. 2009 [R1] | 803 | 2130 |  | - | - |  | 5.51±1.25 | 5.65±1.24 |  | 3.53±1.09 | 3.63±1.09 |  | 1.23±0.35 | 1.23±0.35 |
| Aung et al. 2011 [R2] | 66 | 235 |  | 1.29±1.12 | 1.16±1.14 |  | 5.11±2.30 | 5.19±1.17 |  | 2.75±0.97 | 2.94±0.81 |  | 1.63±0.40 | 1.79±0.52 |
| Aung et al. 2011 (2) [R2] | 70 | 268 |  | 1.03±0.60 | 1.05±0.67 |  | 4.93±1.17 | 4.98±1.25 |  | 2.91±0.88 | 3.0±0.98 |  | 1.77±0.41 | 1.75±0.43 |
| Aung et al. 2011 (3) [R2] | 66 | 233 |  | 1.15±1.16 | 1.30±1.05 |  | 5.41±1.05 | 5.23±1.19 |  | 3.14±0.90 | 2.98±0.84 |  | 1.74±0.47 | 1.64±0.40 |
| Aung et al. 2011 (4) [R2] | 81 | 264 |  | 0.95±0.60 | 1.05±0.94 |  | 4.86±1.09 | 4.90±1.06 |  | 2.82±0.98 | 2.86±0.89 |  | 1.72±0.49 | 1.79±0.69 |
| Liu et al. 2019 [R3] | 37 | 102 |  | 1.83±1.24 | 1.95±1.34 |  | 5.60±0.88 | 5.42±0.80 |  | 3.32±0.49 | 3.44±0.59 |  | 1.21±0.22 | 1.19±0.21 |
| Liu et al. 2019 (2) [R3] | 67 | 98 |  | 1.35±0.86 | 2.33±0.72 |  | 3.93±1.46 | 4.09±1.21 |  | 3.09±0.59 | 3.11±0.77 |  | 1.29± 0.41 | 1.31±0.34 |
| Park et al. 2011 [R4] | 2380 | 5220 |  | 1.70±1.04 | 1.79±1.11 |  | - | - |  | 2.95±0.80 | 3.0±0.82 |  | 1.16±0.24 | 1.16±0.27 |
| Lan et al. 2013 [R5] | 212 | 346 |  | 1.84±1.12 | 2.06±1.60 |  | 5.23±1.02 | 5.25±1.01 |  | 3.20±0.82 | 3.29±0.82 |  | 1.29±0.35 | 1.27±0.28 |
| Lan et al. 2013 (2) [R5] | 153 | 303 |  | 1.55±0.95 | 1.49±0.69 |  | 5.29±0.99 | 5.34±0.94 |  | 3.11±0.82 | 3.12±0.77 |  | 1.52±0.32 | 1.56±0.36 |
| Xu et al. 2013 [R6] | 86 | 201 |  | 1.86±0.91 | 2.01±1.07 |  | 4.27±1.01 | 4.46±1.11 |  | 2.15±1.00 | 2.23±1.19 |  | 1.08±0.23 | 1.08±0.24 |
| Xu et al. 2013 (2) [R6] | 67 | 129 |  | 1.68±1.03 | 2.08±1.10 |  | 4.16±0.94 | 4.36±0.99 |  | 1.94±1.03 | 2.1±0.97 |  | 1.15±0.24 | 1.11±0.25 |
| Wang et al. 2015 [R7] | 936 | 1875 |  | 1.56±0 | 1.66±0.02 |  | 4.38±0 | 4.46±0.02 |  | 2.59±0 | 2.68±0 |  | 1.22±0 | 1.20±0.01 |
| Kathiresan et al. 2008 [R8] [R8] | 1218 | 3753 |  | 1.29±0.67 | 1.39±0.83 |  | - | - |  | 4.11±0.96 | 4.2±1 |  | 1.42±0.39 | 1.39±0.36 |

*TRIB1*: tribbles homolog 1; TG: triglycerides; TC: total cholesterol; LDL-C: low-density lipoprotein cholesterol; HDL-C: high-density lipoprotein cholesterol.

**Table S8.** Plasma lipid levels by the genotypes of *TRIB1* rs2954029 variant.

| **First author, reference** | **Number** | |  | **TG, mmol/L** | |  | **TC, mmol/L** | |  | **LDL-C, mmol/L** | |  | **HDL-C, mmol/L** | |
| --- | --- | --- | --- | --- | --- | --- | --- | --- | --- | --- | --- | --- | --- | --- |
|  | **TT** | **TA+AA** |  | **TT** | **TA+AA** |  | **TT** | **TA+AA** |  | **TT** | **TA+AA** |  | **TT** | **TA+AA** |
| Liu et al. 2019 [R3] | 40 | 106 |  | 1.83±1.21 | 1.95±1.35 |  | 5.54±0.84 | 5.44±0.82 |  | 2.88±0.54 | 3.23±0.51 |  | 1.19±0.24 | 1.20±0.20 |
| Liu et al. 2019 (2) [R3] | 69 | 106 |  | 1.28±0.54 | 1.36±0.87 |  | 4.03±1.47 | 4.57±1.20 |  | 3.08± 0.59 | 3.12±0.76 |  | 1.27±0.40 | 1.32±0.34 |
| Shen et al. 2013 [R11] | 1245 | 2249 |  | - | - |  | 4.03±0.89 | 4.13±0.78 |  | 2.50±0.81 | 2.58±0.73 |  | 1.39±0.31 | 1.41±0.32 |
| Liu et al. 2019 (3) [R12] | 55 | 101 |  | - | - |  | - | - |  | 3.08 ±0.59 | 3.12 ±0.76 |  | - | - |
| Ikeoka et al. 2014 [R13] | 212 | 646 |  | - | - |  | 4.88±0.83 | 4.94±0.83 |  | 2.94±0.72 | 2.98±0.74 |  | 1.40±0.34 | 1.45±0.36 |
| Ikeoka et al. 2014 (2) [R13] | 351 | 1132 |  | - | - |  | 5.28±0.87 | 5.35±0.85 |  | 3.18±0.78 | 3.24±0.77 |  | 1.60±0.34 | 1.59±0.36 |
| Li et al. 2017 [R14] | 276 | 809 |  | 1.14 ±0.53 | 1.18±0.61 |  | - | - |  | - | - |  | - | - |

*TRIB1*: tribbles homolog 1; TG: triglycerides; TC: total cholesterol; LDL-C: low-density lipoprotein cholesterol; HDL-C: high-density lipoprotein cholesterol.

**
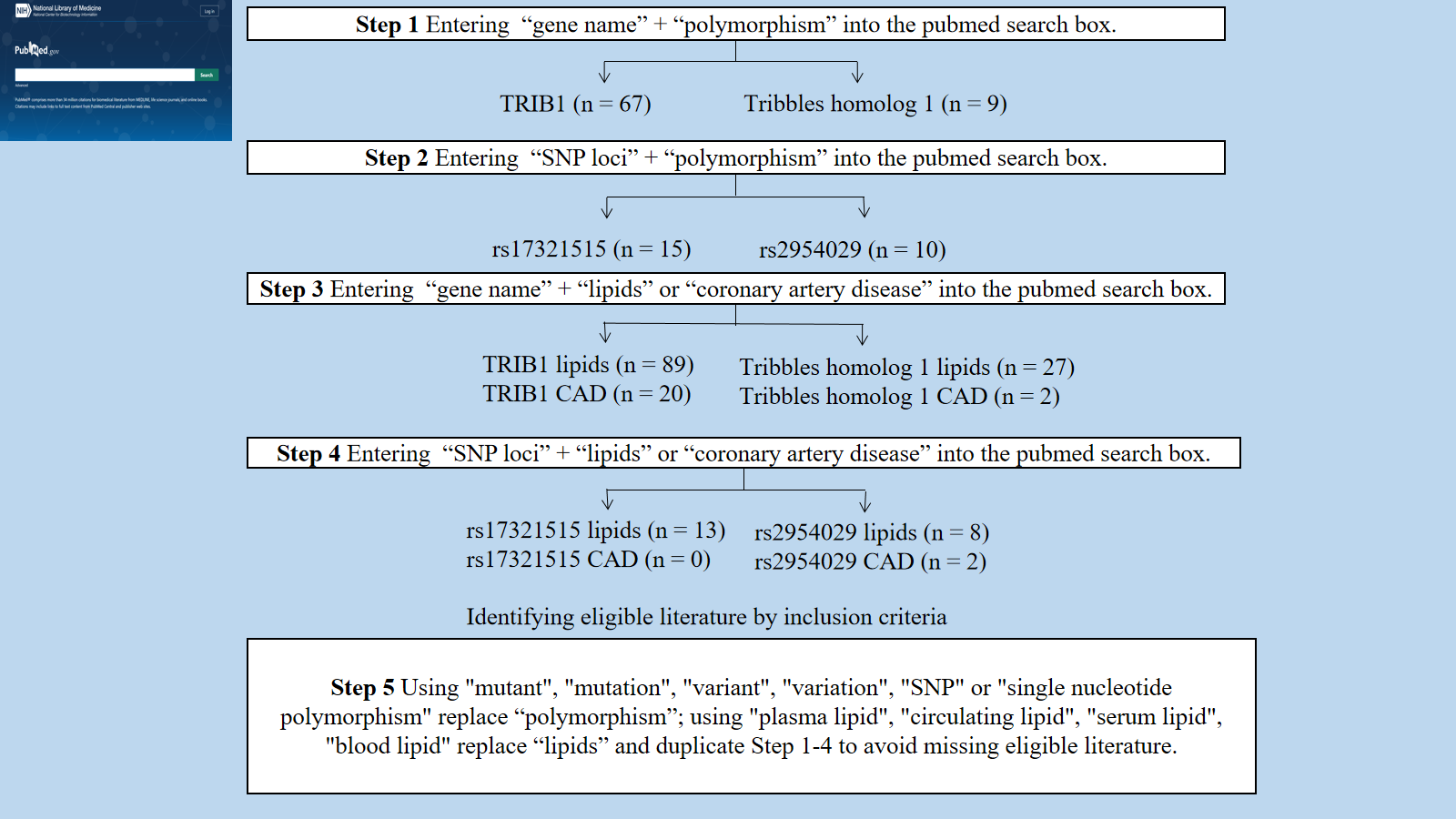
**

**Figure S1.** The full electronic search strategy in Pubmed.


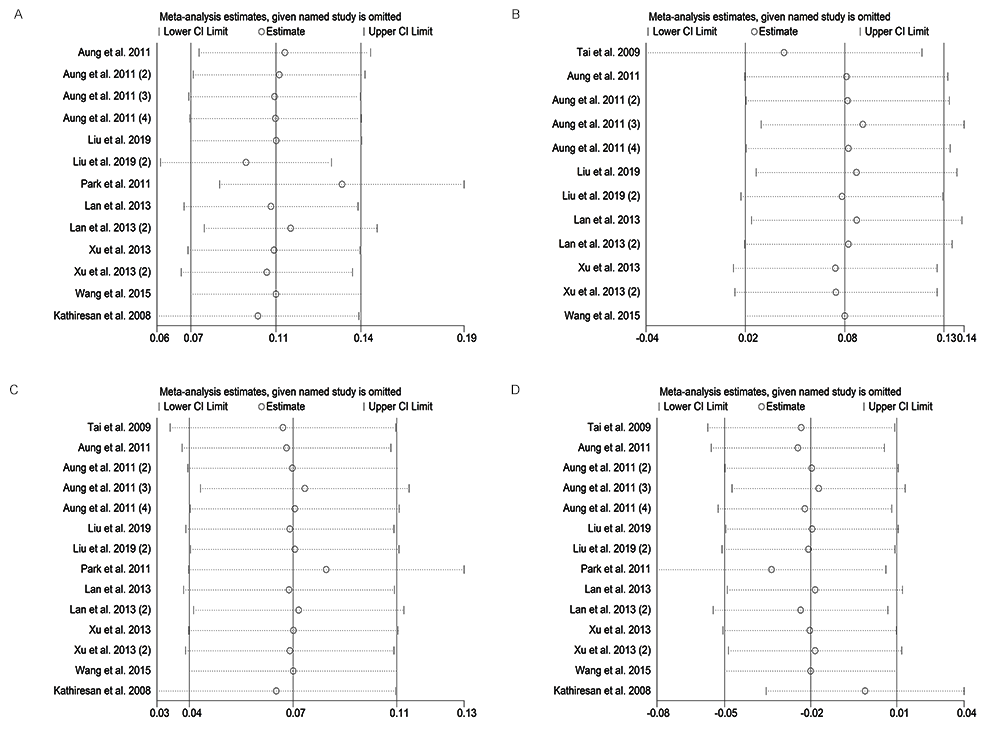


**Figure S2.** Sensitivity analysis of *TRIB1* rs17321515 variant with lipid profile. Open circle is SMD, parallel lines represent 95% CI (A: rs17321515 with TG levels; B: rs17321515 with TC levels; C: rs17321515 with LDL-C levels; D: rs17321515 with HDL-C levels).


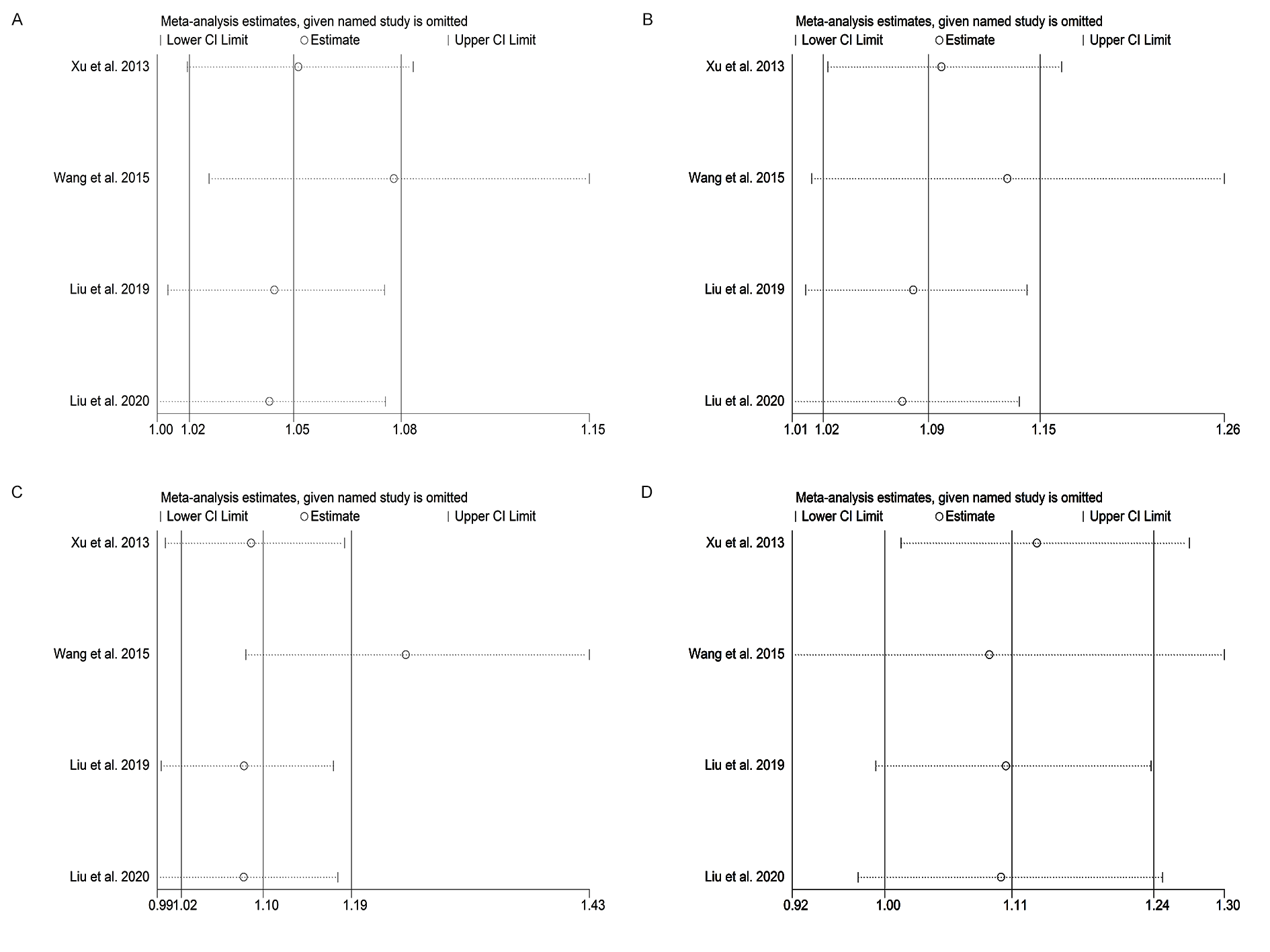


**Figure S3.** Sensitivity analysis of *TRIB1* rs17321515 variant with CAD risk. Open circle is SMD, parallel lines represent 95% CI [A: allelic model (A vs G); B: additive model (AA vs GG); C: dominant model (GA+AA vs GG ); D: recessive model (GG+GA vs AA)].


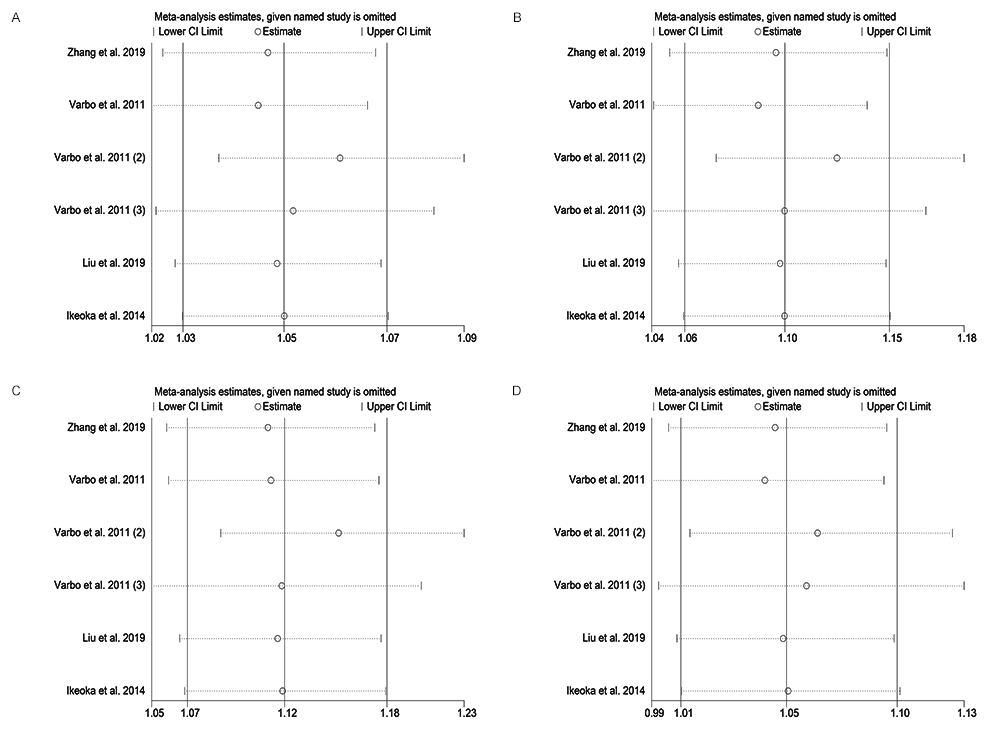


**Figure S4.** Sensitivity analysis of *TRIB1* rs2954029 variant with CAD risk. Open circle is SMD, parallel lines represent 95% CI [A: allelic model (A vs T); B: additive model (AA vs TT); C: dominant model (TA+AA vs TT ); D: recessive model (TT+TA vs AA)].

**
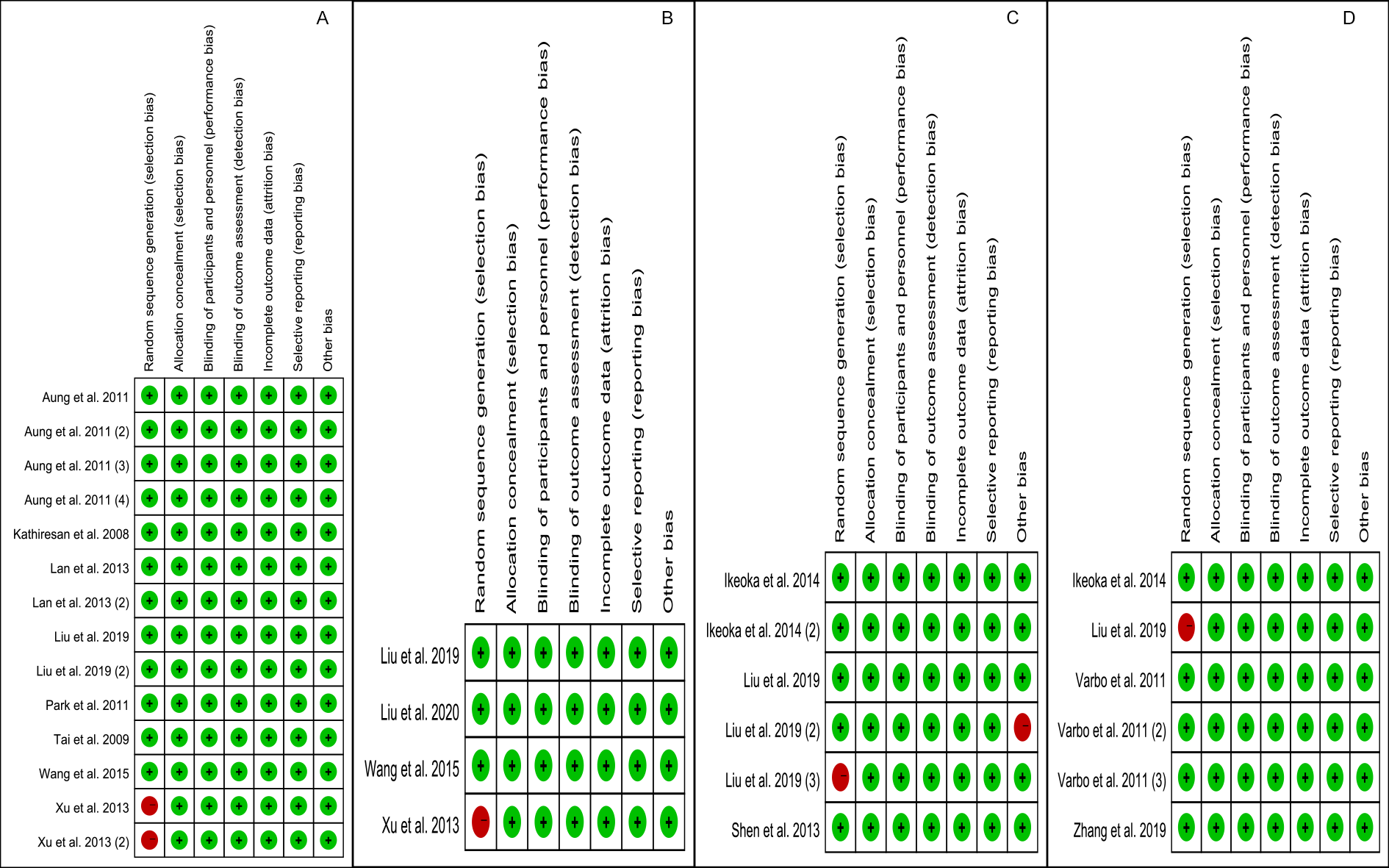
**

**Figure S5.** Risk bias plot of *TRIB1* variant with LDL-C levels and CAD risk. (A: rs17321515 with LDL-C levels; B: rs17321515 with CAD risk; C: rs2954029 with LDL-C levels; D: rs2954029 with CAD risk).


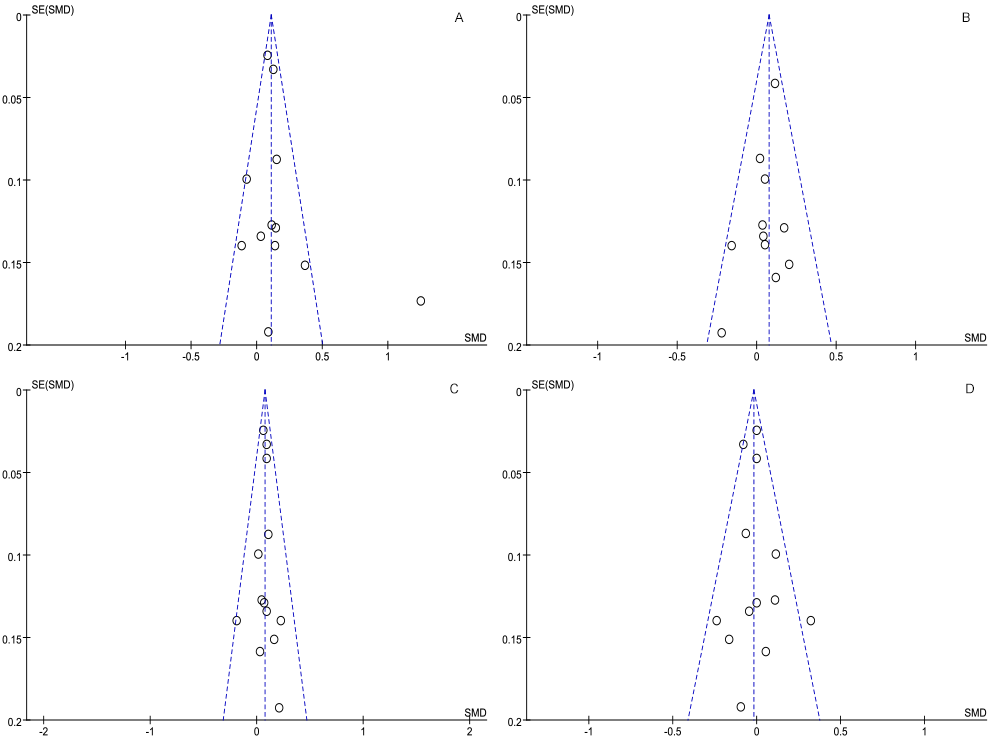


**Figure S6.** Begg’s funnel plot of *TRIB1* rs17321515 variant with lipid profile. Each small circle represents a separate study, the diverging lines represent 95% CI and the central line is SMD. [A: rs17321515 with TG levels (*P* = 0.32); B: rs17321515 with TC levels (*P* = 0.17); C: rs17321515 with LDL-C levels (*P* = 0.92); D: rs17321515 with HDL-C levels (*P* = 0.72)]


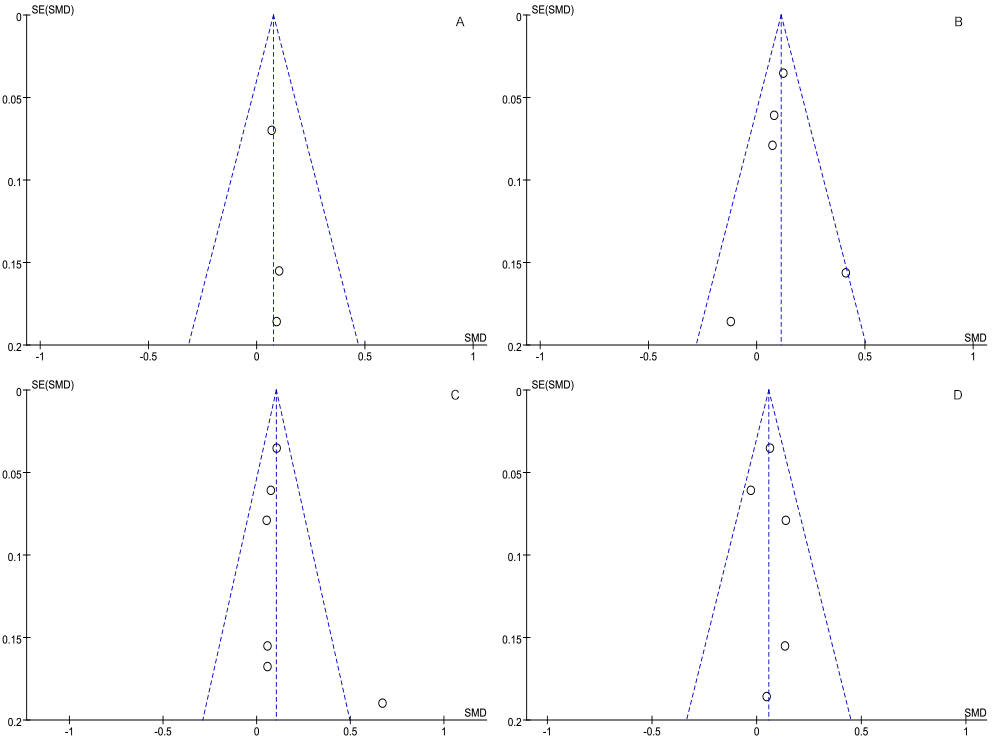


**Figure S7.** Begg’s funnel plot of *TRIB1* rs2954029 variant with lipid profile. Each small circle represents a separate study, the diverging lines represent 95% CI and the central line is SMD. [A: rs2954029 with TG levels (*P* = 0.27); B: rs2954029 with TC levels (*P* = 1); C: rs2954029 with LDL-C levels (*P* = 0.48); D: rs2954029 with HDL-C levels (*P* = 0.77)]


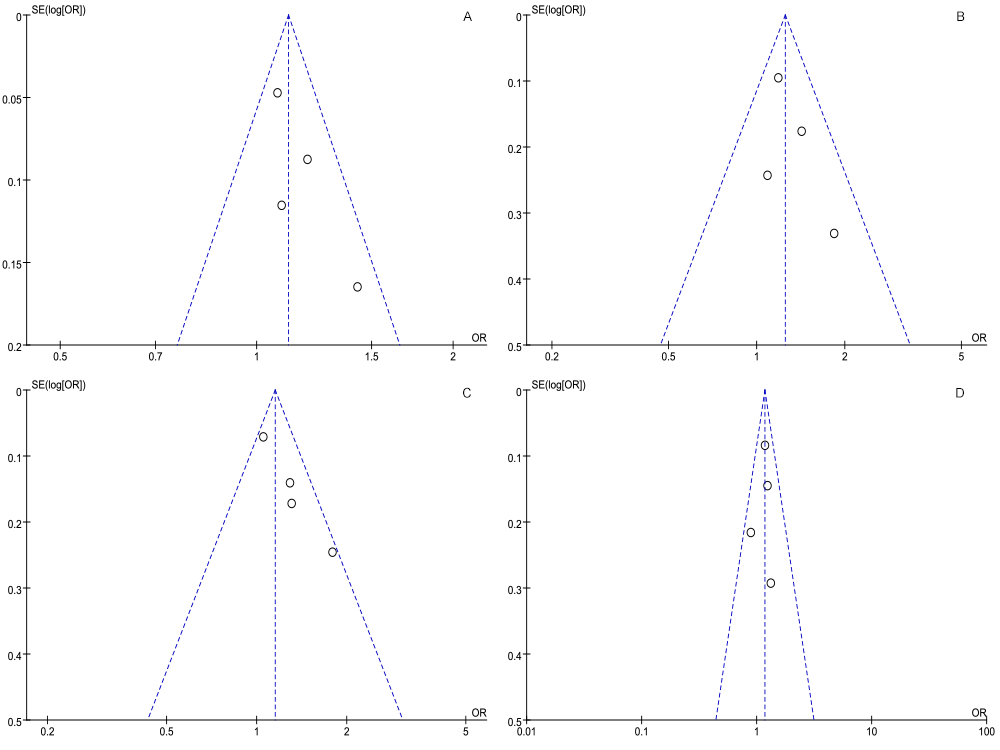


**Figure S8.** Begg’s funnel plot of *TRIB1* rs17321515 variant with CAD risk. Each small circle represents a separate study, the diverging lines represent 95% CI and the central line is SMD. [A: allelic model (*P* = 0.18); B: additive model (*P* = 0.32); C: dominant model (*P* = 0.17); D: recessive model (*P* = 0.73)].


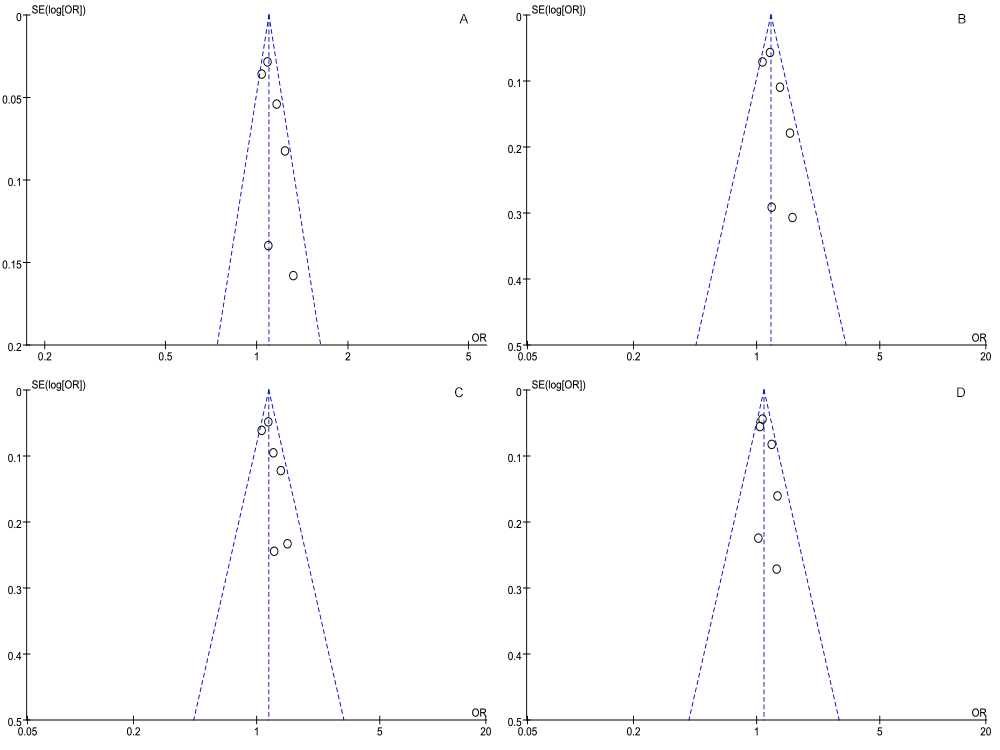


**Figure S9.** Begg’s funnel plot of *TRIB1* rs2954029 variant with CAD risk. Each small circle represents a separate study, the diverging lines represent 95% CI and the central line is SMD. [A: allelic model (*P* = 0.20); B: additive model (*P* = 0.30); C: dominant model (*P* = 0.17); D: recessive model (*P* = 0.20)].
